# Frequency, breed predispositions and other demographic risk factors for diagnosis of hypothyroidism in dogs under primary veterinary care in the UK

**DOI:** 10.1186/s40575-022-00123-8

**Published:** 2022-10-10

**Authors:** Dan G. O’Neill, Janine Su Pheng Khoo, Dave C. Brodbelt, David B. Church, Camilla Pegram, Rebecca F. Geddes

**Affiliations:** 1 Pathobiology and Population Sciences, The Royal Veterinary College, North Mymms, Hawkshead Lane, Hatfield, AL9 7TA Herts UK; 2 Clinical Science and Services, The Royal Veterinary College, Hawkshead Lane, North Mymms, Hatfield, AL9 7TA Herts UK

**Keywords:** Breed, Electronic patient record, Epidemiology, Primary-care, VetCompass

## Abstract

**Background:**

Hypothyroidism is a commonly diagnosed endocrinopathy in dogs. This study aimed to investigate the frequency and risk factors for diagnosis of hypothyroidism in UK primary-care practice. Dogs diagnosed with hypothyroidism were identified by searching electronic patient records of primary-care practices participating in VetCompass. A cohort study design estimated one-year (2016) period prevalence and incidence risk for hypothyroidism. Multivariable binary logistic regression models were used to evaluate associations between demographic risk factors and hypothyroidism.

**Results:**

From 905,553 dogs, 2,105 dogs were recorded with diagnosed hypothyroidism in 2016; 359 incident and 1,746 pre-existing, giving an annual prevalence of 0.23% (95% CI 0.22–0.24) and annual incidence risk of 0.04% (95% CI 0.04–0.04). Multivariable logistic regression identified 24 predisposed and nine protected breeds. Standard Doberman pinscher (odds ratio [OR] = 17.02, 95% CI 12.8–22.64), Tibetan terrier (11.25, 95% CI 8.27–15.32) and boxer (10.44, 95% CI 8.66–12.58) breeds showed high predisposition. Pug (OR 0.29, 95% CI 0.09–0.89), Yorkshire terrier (OR 0.38, 95% CI 0.24–0.59), Shih-tzu (OR 0.38, 95% CI 0.23–0.64) and Jack Russell terrier (OR 0.40, 95% CI 0.29–0.54) were the most protected breeds. Overall, being a purebred dog, being insured, having bodyweight above the breed-sex mean, increasing age, being neutered and rising adult bodyweight also showed increased odds being a dog living with a diagnosis of hypothyroidism.

**Conclusions:**

Several strong breed predispositions for diagnosis of hypothyroidism were identified that can assist with disorder prioritisation in ongoing efforts to improve breed health. Other risk factors were also identified that can assist veterinary surgeons during clinical work-up of suspected cases. Identification of novel evidence for protected breeds provides useful information for research into genetic mechanisms.

## Plain English summary

Hypothyroidism is a commonly diagnosed hormonal disorder in dogs, resulting from a deficiency of thyroid hormone. Affected dogs often show weight gain, lethargy, hair loss and intolerance to cold temperatures. Historically, several dog breeds such as Doberman pinscher, retrievers, schnauzers, Irish setters, Shetland sheepdogs and dachshunds were reported at higher risk of hypothyroidism but there is limited recent work on the frequency and breed risks. This study aimed to explore anonymised clinical records from first opinion veterinary clinics in the UK to report the frequency of diagnosis of hypothyroidism and to identify breeds at most and least risk.

The study included anonymised clinical records on 905,553 dogs across the UK. Overall, 2,105 cases diagnosed with hypothyroidism were identified, showing that 0.23% are affected with hypothyroidism each year in the UK. Across all breeds in the UK, 24 breeds showed increased risk of hypothyroidism and nine breeds showed reduced risk. Compared with crossbred dogs, the breeds with the highest risk of hypothyroidism were Standard Doberman pinscher (17.02 times risk), Tibetan terrier (11.25 times risk), Boxer(10.44 times risk), Alaskan malamute (9.71 times risk), American cocker spaniel (8.64 times risk), and Shetland sheepdog (8.25 times risk). The breeds with the lowest risk were West Highland white terrier (0.40 times risk), Jack Russell terrier (0.40 times risk), Shih-tzu (0.38 times risk), Yorkshire terrier (0.38 times risk), Pug (0.29 times risk), and French bulldog (0.27 times risk). Dogs weighing above average for their breed-sex had 2.06 times the risk of hypothyroidism compared with dogs weighing below their breed-sex average. The risk of hypothyroidism rose as dogs got older.

Based on veterinary information from a large sample of dogs, hypothyroidism is shown to be diagnosed in around one in 400 dogs each year in the UK. The risk of diagnosis of hypothyroidism varied widely across breeds. Awareness of this information can help UK dog owners and veterinary professionals to spot cases of hypothyroidism earlier and therefore to commence treatment sooner to protect the welfare of affected dogs.

## Background

Hypothyroidism is a commonly diagnosed endocrinopathy in dogs, resulting from a deficiency in the thyroid hormones triiodothyronine (T_3_) and thyroxine (T_4_). Primary acquired hypothyroidism due to irreversible destruction of the thyroid glands accounts for the majority of cases [1, 2]. Histopathologically, two forms of primary hypothyroidism are found in most dogs: lymphocytic thyroiditis and non-inflammatory idiopathic follicular atrophy. Most estimates indicate a 1:1 ratio of these two diagnoses as the origin for clinical hypothyroidism in dogs [1]. The most common autoantibody found in sera of hypothyroid dogs with lymphocytic thyroiditis is thyroglobulin (TgAA) [3], although the prevalence of TgAA-positive hypothyroidism varies greatly between breeds [1]. Deficiency of thyroid hormones can result in numerous clinical signs, most commonly associated with metabolic and dermatological effects [4]. Hypothyroidism can therefore impact significantly on quality of life, and in severe, albeit rare cases, can result in myxedema coma, a life-threatening metabolic condition [5]. Studies from referral and teaching hospital populations have estimated the prevalence of canine hypothyroidism at 0.2–0.8% of referral animals [4, 6]. However, this condition is frequently managed entirely within the primary-care practice setting, where data on the frequency of the condition have been largely lacking.

Several risk factors have been reported for hypothyroidism in dogs. Doberman pinscher, retrievers, schnauzers, Irish setters, Shetland sheepdogs and dachshunds are reported as over-represented [4, 6–8]. Increased risk is reported in middle-aged to older dogs, with a mean reported age at first diagnosis of approximately 7 years [4, 6]. However, earlier age at first diagnosis is reported in some high risk breeds [8] and dogs with TgAA-positive hypothyroidism [1]. Neutering has been associated with increased risk of hypothyroidism in some studies [6, 8] although this was not supported by one UK study [4]. However, the validity of these risk factors for extrapolation to the current UK general population of dogs is questionable because many of these studies were performed in the USA, explored referral or teaching hospital study cohorts that were often from the 1990s or earlier, and most of the studies applied univariable analyses where results may be heavily biased by confounding effects [9–11]. In addition, the breed structure of UK dog breeds has changed substantially over recent years, with rising popularity of certain brachycephalic breeds and designer crossbreeds [11, 12]. Further, there is limited published information on breeds with reduced risk of hypothyroidism (i.e. protected to the condition) [13] although one study reported that German Shepherd Dogs and crossbreeds were underrepresented for hypothyroidism [8]. Major histocompatibility complex DLA class II genotypes are reported to associate with hypothyroidism in certain breeds [14, 15] and genome wide association and whole genome sequencing studies are starting to explore additional risk loci in canine hypothyroidism [16, 17]. Therefore, further investigation of predisposed and protected breeds in the UK is warranted to inform genetic studies and assist clinical decision-making by veterinary surgeons for suspected cases.

This study aimed to estimate annual period prevalence and incidence risk during 2016 for diagnosis of hypothyroidism for dogs within UK primary-care practice, and to investigate risk factors for having a diagnosis of hypothyroidism. The study placed particular emphasis on exploring breed-related risk factors that both predispose for and protect against hypothyroidism.

## Methods

The study population included dogs under primary veterinary care in the VetCompass Programme during 2016. Dogs under veterinary care had either a) ≥ 1 electronic patient record (EPR) (free-text clinical note, treatment or bodyweight) recorded during 2016 or b) ≥ 1 EPR recorded during both 2015 and 2017. VetCompass collates de-identified EPR data from primary-care veterinary practices in the UK for epidemiological research [18].

A cohort study design was used. Power calculations estimated that at least 52,235 dogs were needed to estimate prevalence for a disorder occurring in 0.35% of dogs with 0.05% acceptable margin of error at a 95% confidence level from a national UK population of 8 million dogs [19, 20]. Ethics approval was obtained from the Royal Veterinary College Social Sciences Research Ethical Review Board on 2 October 2018 (reference SR2018-1652).

The hypothyroidism case definition required evidence supporting a final diagnosis of hypothyroidism at any date up to 31 December 2016, or that levothyroxine supplementation was prescribed before 31 December 2016 with supporting evidence that this was intended to treat hypothyroidism. The consistent criterion applied for cases of hypothyroidism was that this diagnosis was made in the primary care setting and therefore reflects the clinical opinion of a veterinary surgeon registered with the Royal College of Veterinary Surgeons (RCVS) [21]. The study did not apply any additional laboratory or other specific diagnostic criteria to further sub-classify diagnosed cases based on clinical certainty or to ensure these cases met some external diagnostic consensus. Case-finding followed a two-step process. In step one, all clinical records to 31st December 2016 were searched for candidate (potential) hypothyroidism cases using the clinical free-text field: (hypothy*, hypoT4*, hypoT, “thyroid dz”, “thyroid dx”, undera* thy*, “L-thyroxine”, TgAA, TSH, with fuzziness to allow for misspellings) and treatment fields search terms (solox*, thyf*, levo*, leven*, forthy*, thyr*, canitr*, TSH). In step two, these candidate cases were randomly ordered and the clinical notes of all 22,401 candidate animals were manually reviewed to evaluate against the hypothyroidism case definition and were required to have evidence of a final diagnosis by an RCVS-registered veterinary surgeon for inclusion as a case in the analysis. Date at first diagnosis was extracted for confirmed cases.

Breed descriptive information entered by the participating practices was cleaned and mapped to a VetCompass breed list derived and extended from the VeNom Coding breed list that included both recognised purebred breeds and also designer breed terms [22]. A *purebred status* variable categorised dogs of recognisable breeds as ‘purebred’, dogs with contrived names generated from two or more purebred breed terms as ‘designers’ and dogs recorded as mixes of breeds but without a contrived name as ‘crossbred’ [23]. A *breed* variable included individual pure breeds and designer hybrids represented by ≥ 3000 dogs in the overall study population or with ≥ 10 diagnosed hypothyroidism cases, a grouping of all remaining breeds and of general crossbred dogs. Given the current and growing focus on health issues related to conformation in dogs [24, 25], breeds were further characterised by skull shape (dolichocephalic, mesocephalic, brachycephalic, uncategorised) for exploratory analysis of associations between skull shape and hypothyroidism. A *Kennel Club breed group* variable classified breeds recognised by the UK Kennel Club into their relevant breed groups (Gundog, Hound, Pastoral, Terrier, Toy, Utility, Working, non-Kennel Club recognised) [23].

Neuter and insurance status were defined by the final available EPR value. Adult bodyweight described the mean of all bodyweight (kg) values recorded after 18 months. Mean adult bodyweight was generated for all breed/sex combinations that had adult bodyweight available for at least 100 dogs and was used to categorise individual dogs as “at or above the breed/sex mean”, “below the breed/sex mean” and “unspecified”. Adult bodyweight was classified as unspecified for dogs aged over 18 months that did not have a recorded adult bodyweight or because these dogs had not yet reached 18 months of age. Age (years) was defined for cases and non-cases at December 31, 2016 (the final date in 2016 that the clinical status of these dogs was known).

Statistical analyses were conducted using Stata Version 16 (Stata Corporation). One-year (2016) period prevalence and one-year incidence risk (2016) with 95% confidence intervals (CI) were reported. The CI estimates were derived from standard errors based on approximation to the binomial distribution [26]. Risk factor analysis included all confirmed cases as cases and all animals not screened as candidate cases as non-cases. Binary logistic regression modelling evaluated univariable associations between risk factors (*breed, skull shape, purebred status, Kennel Club recognised breed, Kennel Club breed group, adult bodyweight, bodyweight relative to breed/sex mean, age, sex, neuter* and *insurance*) and an outcome of being a dog living with a diagnosis of hypothyroidism during 2016. Because breed was a factor of primary interest for the study, variables that derived from the breed information and therefore were highly correlated with breed (*skull shape, purebred status, Kennel Club recognised breed* and *Kennel Club breed group*) were excluded from initial breed multivariable modelling. Instead, each of these variables individually replaced the *breed* variable in the main final breed-focused model to evaluate their effects after taking account of the other variables. *Adult bodyweight* (a defining characteristic of individual breeds) replaced breed and *bodyweight relative to breed/sex mean* in the final breed-focused model. Risk factors with liberal associations in univariable modelling (*P* < 0.2) were taken forward for multivariable evaluation. Model development used manual backwards stepwise elimination. Clinic attended was evaluated as a random effect and pair-wise interaction effects with biological relevance were evaluated for the final model variables [10]. Biological relevance was defined as statistically significant effects that are likely to have a noteworthy impact on health or survival and that may alter how decisions for a specific problem are taken [27]. The area under the ROC curve and the Hosmer–Lemeshow test were used to evaluate the quality of the model fit and discrimination of the final breed-focused non-random effect multivariable model [10, 28]. Statistical significance was set at *P* < 0.05.

### Results

#### Frequency and demography

**Fig. 1:**
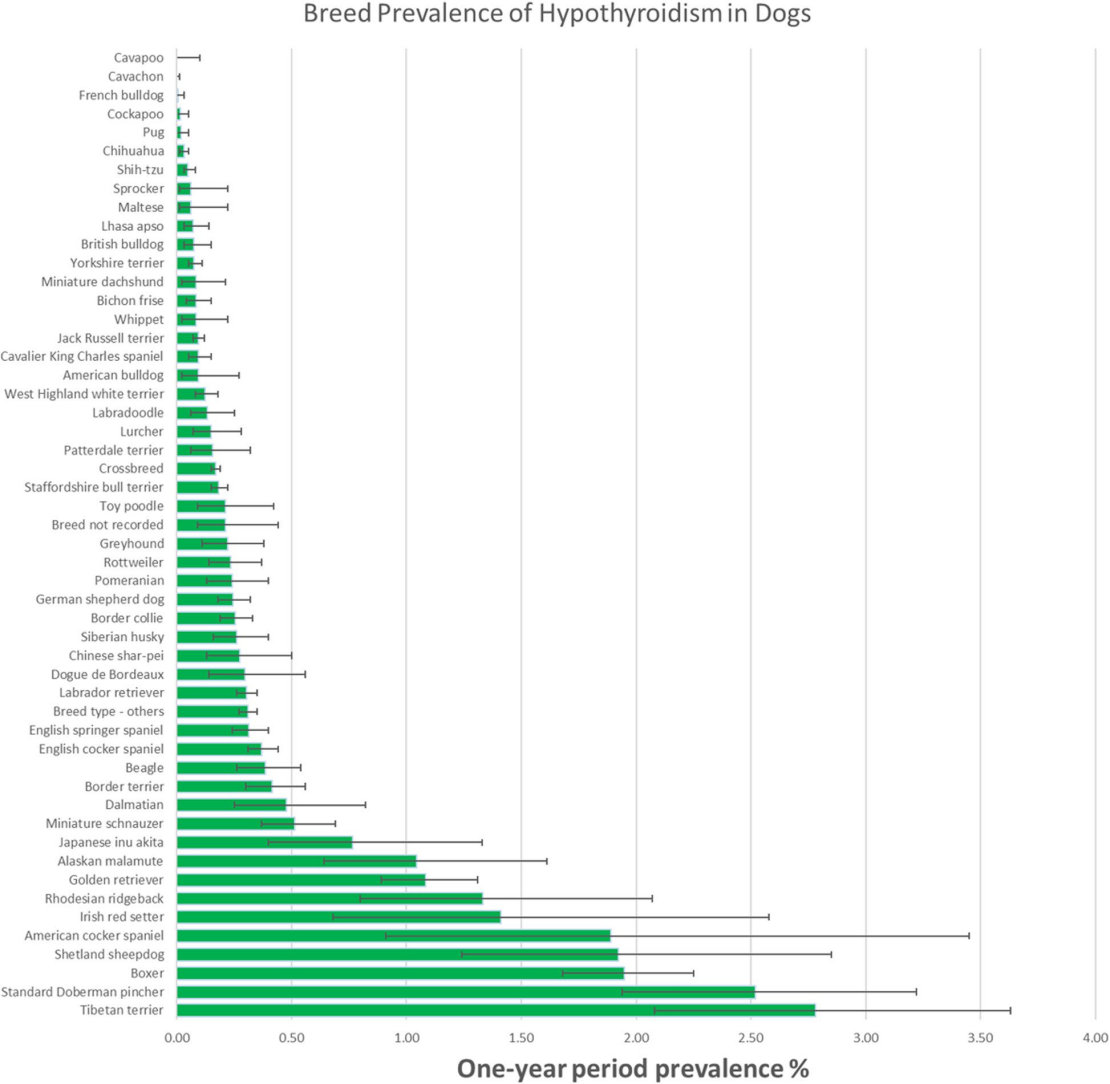
One-year (2016) period prevalence (percentage) of hypothyroidism in dog breeds under primary veterinary care in the VetCompass Programme in the UK. The horizontal bars represent 95% confidence intervals

From a study population of 905,553 dogs under primary veterinary care in 2016 at 887 veterinary clinics, 2,105 diagnosed hypothyroidism cases were confirmed in 2016, giving an annual prevalence of 0.23% (95% CI: 0.22–0.24) among the primary care dog population. Of these dogs, 359 were first diagnosed during 2016, giving an annual incidence risk of 0.04% (95% CI 0.04–0.04). The one-year period prevalence of all cases that existed in 2016 regardless of date of first diagnosis varied widely between the breeds in the study (Fig. 1).

**Fig. 2:**
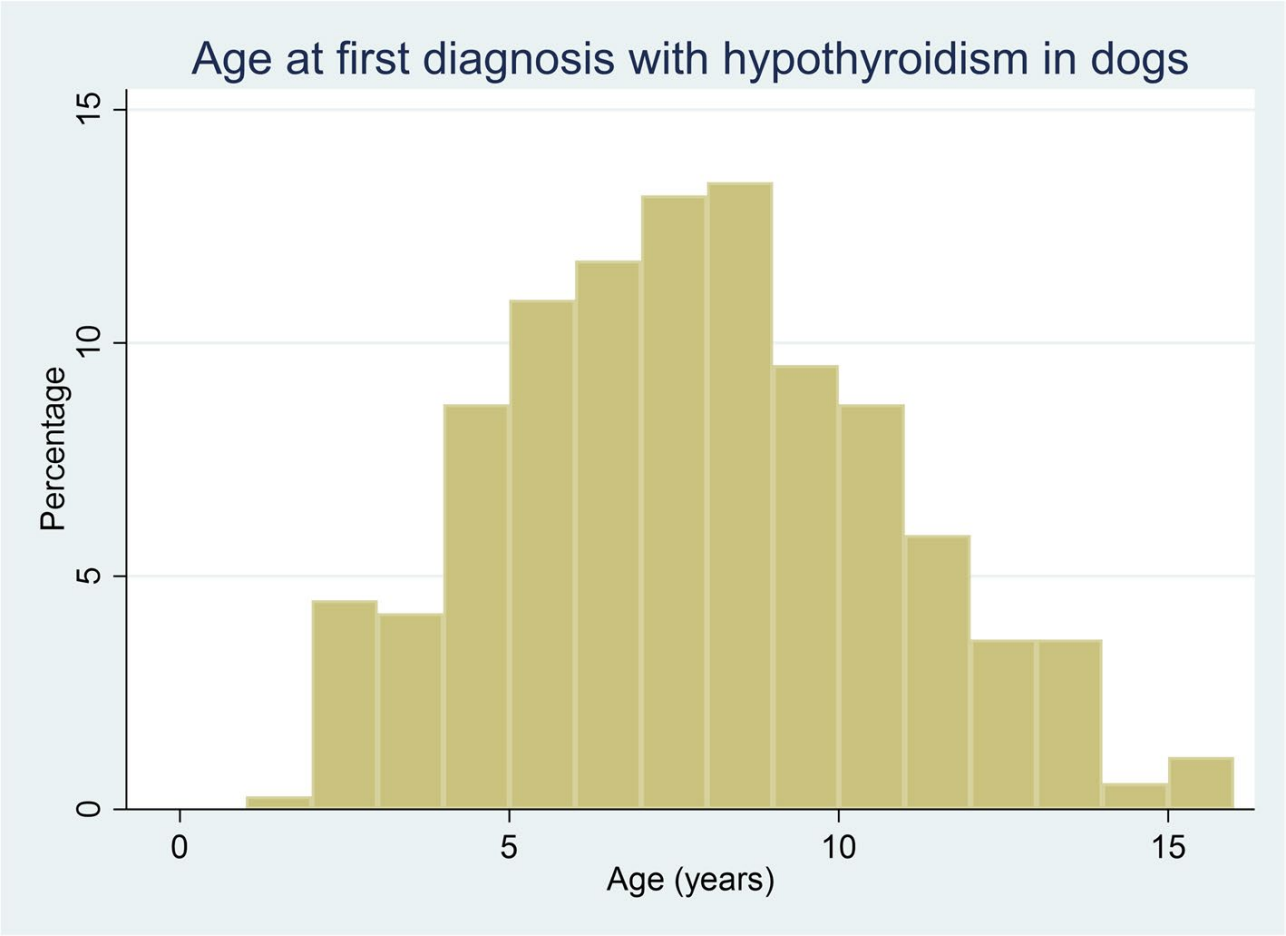
Age (years) at first diagnosis of hypothyroidism in dog breeds under primary veterinary care in the VetCompass Programme in the UK. (*n* = 357)

Of the diagnosed hypothyroidism cases with data available for that variable, 1,734 (82.65%) were purebred, 1,056 (50.31%) were female and 1,468 (69.94%) were neutered. Dogs with hypothyroidism had a median adult bodyweight of 25.63 kg (IQR: 15.16–35.07, range 2.65–82.20). The most common breeds among the hypothyroidism cases were crossbreed (*n* = 332, 15.77%), boxer (184, 8.74%), Labrador retriever (182, 8.65%) and English cocker spaniel (122, 5.80%) (Table 1). The median age at first diagnosis of hypothyroidism in 2016 (*n* = 357) was 7.65 years (IQR 5.65–9.89, range 1.43–15.65) (Fig. 2).

**Table 1:** Descriptive and univariable logistic regression results for breed as a risk factor for hypothyroidism during 2016 in dogs under primary veterinary care in the VetCompass Programme in the UK

Breed	Case No. (% within breed)	Non-case No. (% within breed)	Odds ratio	95% CI	Category *P*-value	Variable *P*-value
Crossbreed	332 (0.17)	194,337 (99.83)	Base			< 0.001
Tibetan terrier	52 (2.78)	1817 (97.22)	16.75	12.46–22.52	< 0.001	
Standard Doberman pinscher	62 (2.52)	2399 (97.48)	15.13	11.5–19.90	< 0.001	
Boxer	184 (1.95)	9258 (98.05)	11.63	9.70–13.95	< 0.001	
Shetland sheepdog	24 (1.92)	1224 (98.08)	11.48	7.56–17.43	< 0.001	
American cocker spaniel	10 (1.89)	519 (98.11)	11.28	5.98–21.28	< 0.001	
Irish red setter	10 (1.41)	698 (98.59)	8.39	4.45–15.80	< 0.001	
Rhodesian ridgeback	19 (1.33)	1406 (98.67)	7.91	4.97–12.60	< 0.001	
Golden retriever	106 (1.08)	9687 (98.92)	6.41	5.14–7.98	< 0.001	
Alaskan malamute	20 (1.04)	1896 (98.96)	6.17	3.92–9.72	< 0.001	
Japanese akita inu	12 (0.76)	1558 (99.24)	4.51	2.53–8.04	< 0.001	
Miniature schnauzer	43 (0.51)	8354 (99.49)	3.01	2.19–4.14	< 0.001	
Dalmatian	13 (0.48)	2707 (99.52)	2.81	1.61–4.90	< 0.001	
Border terrier	40 (0.41)	9611 (99.59)	2.44	1.75–3.38	< 0.001	
Beagle	31 (0.38)	8039 (99.62)	2.26	1.56–3.26	< 0.001	
English cocker spaniel	122 (0.37)	32,953 (99.63)	2.17	1.76–2.67	< 0.001	
English springer spaniel	63 (0.31)	20,145 (99.69)	1.83	1.40–2.40	< 0.001	
Breed—others	262 (0.31)	84,136 (99.69)	1.82	1.55–2.14	< 0.001	
Labrador retriever	182 (0.30)	59,781 (99.70)	1.78	1.49–2.14	< 0.001	
Dogue de Bordeaux	9 (0.30)	3023 (99.70)	1.74	0.90–3.38	0.101	
Chinese shar-pei	10 (0.27)	3639 (99.73)	1.61	0.86–3.02	0.139	
Siberian husky	22 (0.26)	8366 (99.74)	1.54	1.00–2.37	0.050	
Border collie	62 (0.25)	24,326 (99.75)	1.49	1.14–1.96	0.004	
German shepherd dog	52 (0.24)	21,319 (99.76)	1.43	1.07–1.91	0.017	
Pomeranian	15 (0.24)	6206 (99.76)	1.41	0.84–2.37	0.189	
Rottweiler	17 (0.23)	7268 (99.77)	1.37	0.84–2.23	0.207	
Greyhound	12 (0.22)	5444 (99.78)	1.29	0.72–2.30	0.386	
Breed not recorded	7 (0.21)	3279 (99.79)	1.25	0.59–2.64	0.560	
Toy poodle	8 (0.21)	3766 (99.79)	1.24	0.62–2.51	0.543	
Staffordshire bull terrier	97 (0.18)	52,958 (99.82)	1.07	0.85–1.34	0.546	
Patterdale terrier	7 (0.16)	4448 (99.84)	0.92	0.44–1.95	0.830	
Lurcher	9 (0.15)	6013 (99.85)	0.88	0.45–1.70	0.696	
Labradoodle	10 (0.13)	7475 (99.87)	0.78	0.42–1.47	0.446	
West Highland white terrier	23 (0.12)	18,855 (99.88)	0.71	0.47–1.09	0.118	
American bulldog	3 (0.09)	3221 (99.91)	0.55	0.17–1.70	0.296	
Cavalier King Charles spaniel	16 (0.09)	17,242 (99.91)	0.54	0.33–0.90	0.017	
Jack Russell terrier	45 (0.09)	48,525 (99.91)	0.54	0.40–0.74	< 0.001	
Whippet	4 (0.09)	4682 (99.91)	0.50	0.19–1.34	0.168	
Bichon frise	11 (0.08)	13,258 (99.92)	0.49	0.27–0.89	0.018	
Miniature dachshund	4 (0.08)	4824 (99.92)	0.49	0.18–1.30	0.151	
British bulldog	7 (0.07)	9392 (99.93)	0.44	0.21–0.92	0.030	
Yorkshire terrier	21 (0.07)	28,159 (99.93)	0.44	0.28–0.68	< 0.001	
Lhasa apso	9 (0.07)	12,540 (99.93)	0.42	0.22–0.81	0.010	
Maltese	2 (0.06)	3246 (99.94)	0.36	0.09–1.45	0.151	
Sprocker	2 (0.06)	3336 (99.94)	0.35	0.09–1.41	0.140	
Shih-tzu	16 (0.05)	32,894 (99.95)	0.28	0.17–0.47	< 0.001	
Chihuahua	11 (0.03)	36,783 (99.97)	0.18	0.10–0.32	< 0.001	
Pug	3 (0.02)	16,211 (99.98)	0.11	0.03–0.34	< 0.001	
Cockapoo	3 (0.02)	18,249 (99.98)	0.10	0.03–0.30	< 0.001	
French bulldog	1 (0.01)	16,396 (99.99)	0.04	0.01–0.25	0.001	
Cavachon	0 (0.00)	3535 (100.00)	~			
Cavapoo	0 (0.00)	4035 (100.00)	~			

Column percentages shown in brackets

*95% CI* 95% confidence interval

Of the dogs that were not diagnosed with hypothyroidism that had data available on the variable, 653,168 (72.56%) were purebred, 430,652 (47.89%) were female, and 407,497 (45.21%) were neutered. The median adult bodyweight for non-cases was 13.92 kg (IQR: 8.18–25.00, range 0.72–97.20) and the median age was 4.43 years (IQR: 1.87–8.07, range 0.00–20.97). The most common breeds among the non-case dogs were crossbred (*n* = 194,377, 21.51%), Labrador retriever (59,781, 6.62%), Staffordshire bull terrier (52,958, 5.86%) and Jack Russell terrier (48,525, 5.37%) (Table 1).

#### Risk factors

All tested variables were liberally associated with hypothyroidism diagnosis in univariable logistic regression modelling (Tables 1, 2 and 3). The final main breed-focused multivariable model retained five risk factors: *breed, age, bodyweight relative to breed-sex mean, sex-neuter* and *insurance* (Table 4). No biologically relevant interactions were identified. The final model was improved by inclusion of the clinic attended as a random effect (rho: 0.06 indicating that 6% of the variability was accounted for by the clinic attended, *P* < 0.001). The final model showed acceptable model-fit (Hosmer–Lemeshow test statistic: *P* = 0.254) and acceptable discrimination (area under the ROC curve: 0.892).

**Table 2:** Descriptive and univariable logistic regression results for breed-derived risk factors for hypothyroidism during 2016 in dogs under primary veterinary care in the VetCompass Programme in the UK

Variable	Category	Case No. (%)	Non-case No. (%)	Odds ratio	95% CI	Category *P*-value	Variable *P*-value
Purebred status	Crossbred	332 (0.17)	194,337 (99.83)	Base			< 0.001
	Designer	32 (0.06)	52,654 (99.94)	0.36	0.25–0.51	< 0.001	
	Purebred	1,734 (0.26)	653,168 (99.74)	1.55	1.38–1.75	< 0.001	
Kennel Club Recognised Breed	Not recognised	386 (0.15)	261,969 (99.85)	Base			< 0.001
	Recognised	1,712 (0.27)	638,190 (99.73)	1.82	1.63–2.03	< 0.001	
Kennel Club Breed Group	Not Kennel Club recognised breed	386 (0.15)	261,969 (99.85)	Base			< 0.001
	Terrier	248 (0.17)	145,673 (99.83)	1.16	0.96–1.36	0.076	
	Gundog	562 (0.41)	135,111 (99.59)	2.82	2.48–3.21	< 0.001	
	Working	345 (0.88)	38,872 (99.12)	6.02	5.21–6.97	< 0.001	
	Pastoral	173 (0.33)	52,809 (99.67)	2.22	1.86–2.66	< 0.001	
	Utility	195 (0.19)	102,469 (99.81)	1.29	1.09–1.53	0.004	
	Hound	98 (0.31)	31,319 (99.69)	2.12	1.70–2.65	< 0.001	
	Toy	91 (0.07)	131,937 (99.93)	0.47	0.37–0.59	< 0.001	
Skull conformation	Mesocephalic	1,196 (0.29)	416,448 (99.71)	Base			
	Brachycephalic	293 (0.17)	167,140 (99.83)	0.61	0.54–0.69	< 0.001	
	Dolichocephalic	245 (0.35)	69,580 (99.65)	1.23	1.07–1.41	0.004	
	Uncategorised	371 (0.15)	250,270 (99.85)	0.52	0.46–0.58	< 0.001	

Column percentages shown in brackets

*95% CI* 95% confidence interval, *CI* Confidence interval

**Table 3:** Descriptive and univariable logistic regression results for non-breed-related demographic risk factors evaluated for hypothyroidism during 2016 in dogs under primary veterinary care in the VetCompass Programme in the UK

Variable	Category	Case No. (%)	Non-case No. (%)	Odds ratio	95% CI*	Category *P*-value	Variable *P*-value
Adult (> 18 months) bodyweight (kg)	< 10.0	183 (0.09)	213,169 (99.91)	Base			< 0.001
	10.0—< 15.0	259 (0.26)	98,129 (99.74)	3.07	2.54–3.72	< 0.001	
	15.0—< 20.0	207 (0.30)	69,184 (99.70)	3.49	2.86–4.25	< 0.001	
	20.0—< 25.0	211 (0.33)	63,696 (99.67)	3.86	3.17–4.70	< 0.001	
	25.0—< 30.0	246 (0.46)	53,528 (99.54)	5.35	4.42–6.48	< 0.001	
	30.0—< 40.0	436 (0.62)	69,496 (99.38)	7.31	6.15–8.69	< 0.001	
	≥ 40.0	246 (0.94)	26,011 (99.06)	11.02	9.09–13.35	< 0.001	
	Uncategorised	317 (0.10)	310,225 (99.90)	1.19	0.99–1.43	0.061	
Bodyweight relative to breed mean	Lower	557 (0.18)	316,792 (99.82)	Base			< 0.001
	Equal/Higher	1,225 (0.44)	274,288 (99.56)	2.54	2.30–2.81	< 0.001	
	Uncategorised	323 (0.10)	312,358 (99.90)	0.59	0.51–0.67	< 0.001	
Age (years)	< 3.0	9 (0.00)	329,266 (100.00)	0.01	0.01–0.03	< 0.001	
	3.0—< 5.0	82 (0.05)	156,651 (99.95)	0.29	0.22–0.37	< 0.001	
	5.0—< 7.0	231 (0.18)	126,239 (99.82)	Base			< 0.001
	7.0—< 9.0	419 (0.41)	101,832 (99.59)	2.25	1.91–2.64	< 0.001	
	9.0—< 11.0	519 (0.66)	78,466 (99.34)	3.61	3.09–4.22	< 0.001	
	11.0—< 13.0	457 (0.85)	53,158 (99.15)	4.70	4.01–5.51	< 0.001	
	≥ 13.0	371 (0.81)	45,419 (99.19)	4.46	3.79–5.26	0.058	
	Uncategorised	17 (0.14)	12,407 (99.86)	0.75	0.46–1.23	0.250	
Sex-Neuter	Female entire	261 (0.11)	233,566 (99.89)	Base			< 0.001
	Female neuter	795 (0.40)	197,086 (99.60)	3.61	3.14–4.15	< 0.001	
	Male entire	370 (0.14)	259,152 (99.86)	1.28	1.09–1.50	0.002	
	Male neuter	673 (0.32)	209,411 (99.68)	2.88	2.49–3.32	< 0.001	
	Uncategorised	6 (0.14)	4,223 (99.86)	1.27	0.57–2.86	0.561	
Insurance	Non-insured	1,443 (0.18)	786,532 (99.82)	Base			< 0.001
	Insured	662 (0.56)	116,906 (99.44)	3.09	2.81–3.39	< 0.001	

Column percentages shown in brackets

*95% CI* 95% confidence interval

**Table 4:** Final breed-focused random effects multivariable logistic regression model for risk factors associated with hypothyroidism in dogs under primary veterinary care in the VetCompass Programme in the UK. Clinic attended was included as a random effect

Variable	Category	Odds ratio	95% CI	*P*-value	Variable *P*-value
Breed	Crossbreed	Base			< 0.001
	Standard Doberman pinscher	17.02	12.8–22.64	< 0.001	
	Tibetan terrier	11.25	8.27–15.32	< 0.001	
	Boxer	10.44	8.66–12.58	< 0.001	
	Alaskan malamute	9.71	6.11–15.42	< 0.001	
	American cocker spaniel	8.64	4.51–16.55	< 0.001	
	Shetland sheepdog	8.25	5.38–12.65	< 0.001	
	Rhodesian ridgeback	6.80	4.23–10.94	< 0.001	
	Irish red setter	5.64	2.96–10.76	< 0.001	
	Japanese inu akita	4.69	2.61–8.42	< 0.001	
	Dogue de Bordeaux	4.45	2.28–8.7	< 0.001	
	Golden retriever	4.36	3.48–5.45	< 0.001	
	Beagle	3.08	2.12–4.47	< 0.001	
	Miniature schnauzer	3.03	2.19–4.18	< 0.001	
	Pomeranian	2.85	1.69–4.81	< 0.001	
	Chinese shar-pei	2.80	1.48–5.28	0.001	
	Siberian husky	2.53	1.64–3.92	< 0.001	
	Dalmatian	2.24	1.28–3.92	0.005	
	Breed not recorded	2.11	0.96–4.64	0.063	
	English cocker spaniel	2.10	1.7–2.59	< 0.001	
	Breed—others	1.86	1.58–2.2	< 0.001	
	Border terrier	1.86	1.33–2.59	< 0.001	
	Rottweiler	1.64	1.01–2.68	0.047	
	German shepherd dog	1.51	1.12–2.02	0.006	
	English springer spaniel	1.43	1.09–1.87	0.010	
	Labrador retriever	1.40	1.16–1.68	< 0.001	
	Toy poodle	1.30	0.64–2.63	0.469	
	Border collie	1.18	0.9–1.56	0.223	
	British bulldog	1.10	0.52–2.34	0.801	
	Labradoodle	1.06	0.56–2	0.859	
	American bulldog	1.05	0.33–3.27	0.938	
	Staffordshire bull terrier	0.96	0.76–1.2	0.705	
	Patterdale terrier	0.90	0.43–1.91	0.786	
	Sprocker	0.86	0.21–3.48	0.835	
	Lurcher	0.77	0.4–1.5	0.449	
	Greyhound	0.77	0.43–1.38	0.384	
	Maltese	0.67	0.17–2.69	0.571	
	Whippet	0.58	0.22–1.56	0.281	
	Bichon frise	0.50	0.27–0.91	0.024	
	Miniature dachshund	0.49	0.18–1.32	0.159	
	Cockapoo	0.48	0.15–1.51	0.210	
	Chihuahua	0.46	0.25–0.84	0.011	
	Cavalier King Charles spaniel	0.45	0.27–0.75	0.002	
	Lhasa apso	0.44	0.22–0.85	0.014	
	West Highland white terrier	0.40	0.26–0.61	< 0.001	
	Jack Russell terrier	0.40	0.29–0.54	< 0.001	
	Shih-tzu	0.38	0.23–0.64	< 0.001	
	Yorkshire terrier	0.38	0.24–0.59	< 0.001	
	Pug	0.29	0.09–0.89	0.031	
	French bulldog	0.27	0.04–1.96	0.197	
	Cavachon	~			
	Cavapoo	~			
Bodyweight relative to breed-sex mean	Below	Base			< 0.001
	At or above	2.06	1.87–2.29	< 0.001	
	Uncategorised	0.91	0.78–1.06	0.236	
Age (years)	< 3.0 years	0.02	0.01–0.05	< 0.001	< 0.001
	3.0—< 5.0 years	0.32	0.25–0.41	< 0.001	
	5.0—< 7.0 years	Base			
	7.0—< 9.0 years	2.12	1.81–2.5	< 0.001	
	9.0—< 11.0 years	3.24	2.77–3.79	< 0.001	
	11.0—< 13.0 years	4.54	3.86–5.33	< 0.001	
	> or = 13.0 years	5.68	4.8–6.73	< 0.001	
	No age available	1.55	0.91–2.64	0.105	
Sex-Neuter	Female entire	Base			< 0.001
	Female neutered	1.46	1.26–1.69	< 0.001	
	Male entire	1.05	0.89–1.23	0.577	
	Male neutered	1.31	1.13–1.52	< 0.001	
	Unrecorded	1.71	0.73–4.02	0.215	
Insurance	Uninsured	Base			< 0.001
	Insured	2.27	2.05–2.52	< 0.001	

95% CI, 95% confidence interval

~ No cases recorded

After accounting for the effects of the other variables evaluated, 24 breeds showed increased odds of hypothyroidism diagnosis compared with crossbred dogs. The breeds with the highest odds included standard Doberman pinscher (odds ratio [OR] 17.02, 95% CI 12.8–22.64, *P* < 0.001), Tibetan terrier (OR 11.25, 95% CI 8.27–15.32, *P* < 0.001), boxer (OR 10.44, 95% CI 8.66–12.58, *P* < 0.001) and Alaskan malamute (OR 9.71, 95% CI 6.11–15.42, *P* < 0.001). Nine breeds showed reduced odds of hypothyroidism diagnosis compared with crossbreds. The breeds with the lowest odds of hypothyroidism included Pug (OR 0.29, 95% CI 0.09–0.89, *P* = 0.031), Yorkshire terrier (OR 0.38, 95% CI 0.24–0.59, *P* < 0.001), shih-tzu (OR 0.38, 95% CI 0.23–0.64, *P* < 0.001) and Jack Russell terrier (OR 0.40, 95% CI 0.29–0.54, *P* < 0.001). There were also two common breeds without any recorded hypothyroidism diagnosis cases: cavachon and cavapoo.

Dogs that weighed at or above the mean for their breed-sex showed 2.06 (95% CI 1.87–2.29, *P* < 0.001) times the odds compared with dogs weighing below their breed-sex mean. The odds of having hypothyroidism diagnosis rose as dogs aged. Dogs aged 11–13 years had 4.54 (95% CI 3.86–5.33, *P* < 0.001) times the odds compared with dogs aged from 5 to under 7 years. Neutered females (OR 1.46, 95% CI 1.26–1.69, *P* < 0.001) and neutered males (OR 1.31, 95% CI 1.13–1.52, *P* < 0.001) had higher odds than entire females. Insured dogs had 2.27 (95% CI 2.05–2.52, *P* < 0.001) times the odds of diagnosed hypothyroidism compared with uninsured dogs (Table 4).

As described in the methods, breed-derived variables were introduced individually to replace *breed* in the final breed-focused model. Compared with crossbred dogs, purebred dogs had increased odds of diagnosed hypothyroidism (OR 1.49, 95% CI 1.32–1.68, *P* < 0.001). Among the Kennel Club breed groups, Working, Gundog, Hound, Pastoral and Utility showed higher odds while Terrier and Toy showed lower odds of diagnosed hypothyroidism compared with breeds not recognized by the Kennel Club. Compared with breeds with mesocephalic skull conformation, breeds with dolichocephalic (OR 1.35, 95% CI 1.18–1.55, *P* < 0.001) or brachycephalic skull conformations (OR 1.15, 95% CI 1.01–1.31, *P* = 0.037) showed higher odds of diagnosed hypothyroidism. The odds of diagnosed hypothyroidism increased as adult bodyweight increased (Table 5).

**Table 5:** Results for risk factors that directly replaced the breed variable in the final breed-focused random effects multivariable logistic regression model (along with age, *bodyweight relative to breed-sex mean* sex/neuter and insurance status). Adult (> 18 months) bodyweight (kg) replaced the breed and bodyweight relative to breed mean variables in the final breed-focused random effects multivariable logistic regression model. These results report associations between these risk factors and hypothyroidism in dogs under primary veterinary care in the VetCompass Programme in the UK. Clinic attended was included as a random effect

Variable	Category	Odds ratio	95% CI*	*P*-value	Variable *P*-value
Purebred	Crossbred	Base			< 0.001
	Designer	0.84	0.58–1.21	0.342	
	Purebred	1.49	1.32–1.68	< 0.001	
Kennel Club Recognised Breed	Not recognised	Base			< 0.001
	Recognised	1.51	1.35–1.69	< 0.001	
Kennel Club Breed Group	Not Kennel Club recognised breed	Base			< 0.001
	Working	6.55	5.64–7.60	< 0.001	
	Gundog	1.97	1.72–2.24	< 0.001	
	Hound	1.78	1.42–2.22	< 0.001	
	Pastoral	1.67	1.40–2.00	< 0.001	
	Utility	1.55	1.30–1.84	< 0.001	
	Terrier	0.75	0.64–0.88	0.001	
	Toy	0.53	0.42–0.67	< 0.001	
Skull conformation	Mesocephalic	Base			< 0.001
	Dolichocephalic	1.35	1.18–1.55	< 0.001	
	Brachycephalic	1.15	1.01–1.31	0.037	
	Unavailable	0.71	0.63–0.80	< 0.001	
Adult (> 18 months) bodyweight (kg)	< 10.0	Base			< 0.001
	10.0—< 15.0	2.73	2.26–3.30	< 0.001	
	15.0—< 20.0	2.95	2.41–3.60	< 0.001	
	20.0—< 25.0	3.00	2.46–3.66	< 0.001	
	25.0—< 30.0	4.13	3.41–5.01	< 0.001	
	30.0—< 40.0	5.60	4.70–6.67	< 0.001	
	≥ 40.0	9.85	8.10–11.97	< 0.001	
	Unavailable	1.82	1.50–2.21	< 0.001	

*95% CI* 95% confidence interval

## Discussion

This is the first study to report the frequency and demographic risk factors for diagnosed hypothyroidism in dogs under UK primary veterinary care and based on the typical UK primary care standards of diagnosis. Access to clinical data from a primary care perspective allows for exploration of diagnosed caseloads in the wider dog population under primary veterinary care compared to the smaller subset of cases that are promoted for referral care [29]. This paper contributes to the increasing volume of scientific output from primary care practice-based research over the past decade that promotes growing recognition of primary care veterinary medicine as a specialism [18, 30, 31].

One-year period prevalence for diagnosed hypothyroidism was estimated at 0.23%, at the lower end of two previous prevalence estimates from referral and teaching hospital populations of 0.2–0.8% [4, 6]. Annual prevalence includes both cases diagnosed in the current year as well as cases diagnosed in previous years that still survive. With 83% of prevalent hypothyroidism cases in the current study being first diagnosed prior to the year of interest, this suggests a relatively long survival following diagnosis for hypothyroidism. The results for the prevalence values and for the risk factor analysis should therefore be interpreted as representing dogs that continue to live with a diagnosis of hypothyroidism in 2016 rather than reflecting the risk of developing hypothyroidism as a de novo condition. Supporting this important distinction between typical ages for developing disease compared with typical ages of dogs living with disease, the median age at first diagnosis of hypothyroidism in the current study was 7.65 years (very similar to previous reports of 7.2–7.5 years [4, 6]) whereas it was the oldest age band of dogs (aged over 13 years) that had the highest odds of living with a diagnosis of hypothyroidism. Increasing age above 7 years was independently associated with having hypothyroidism, with increasing odds ratios for every advancing age category, which was expected given that hypothyroidism is a life-long, but rarely lifespan-limiting condition. The current study indicated the clinic attended by each dog accounted for 6% of the variability in the diagnoses, suggesting relatively high differential probability of diagnoses between clinics perhaps because of differing levels of clinical specialism and experience, diagnostic resources and economic support for diagnosis. It is increasingly recognised that primary veterinary care involves high levels of complexity within diagnostic pathways and client communication [32]. The epidemiological unit for the current study was the dog but deeper understanding of diagnostic pathways could explore whether these cases presented primarily for signs related to their hypothyroidism or whether the hypothyroidism was detected secondarily during veterinary examination for other reasons. Such information on the nature of these diagnoses could assist to develop improved indices of suspicion across ages and breeds that could enhance disorder discernment [33].

Breed was strongly associated with the odds of diagnosed hypothyroidism in the current study. Standard Doberman pinscher had the highest odds of diagnosed hypothyroidism compared with crossbreds following multivariable analyses (OR = 17.02) and has previously been reported as predisposed in multiple studies [6–8]. Standard Doberman pinschers are reported with an association between TgAA-positive hypothyroidism and the presence of a rare DLA class II haplotype, DLA-DRB1*01,201/DQA1*00,101/DQB1*00,201 [14]. The allele, DQA1*00,101, is also reportedly common in Rhodesian ridgebacks and English setters with hypothyroidism, but is not raised in all predisposed breeds, e.g. Boxers [14]. Interestingly, fewer than 30% of Dobermans with hypothyroidism are TgAA-positive [1, 3], therefore further investigation of genetic loci associated with hypothyroidism in this breed are warranted.

Among the 24 breeds identified with increased odds of diagnosed hypothyroidism in the current study, three breeds demonstrated ultra-predispositions, with over ten-times the odds of diagnosed hypothyroidism compared to crossbred dogs. Of these ultra-predisposed breeds, the Tibetan terrier has not been previously reported at increased risk, yet in the present study had the highest one-year period prevalence of hypothyroidism, with 2.78% of the breed affected. Smaller case numbers in previous clinical studies may have prevented earlier reporting of this predisposition, although the Tibetan terrier was among the top 20 breeds with the highest prevalence of TgAA for cases with serum submitted for investigation of thyroid disease in one study [1].

The current study reports purebred dogs with 1.49 times higher overall odds of diagnosed hypothyroidism compared to crossbred dogs. This is consistent with results from a large cohort of US referral patients where purebred dogs had 1.56 times the odds of hypothyroidism compared to mixed-breed dogs [34]. A purebred predisposition to hypothyroidism was also widely pervasive across that study’s purebred population, with increased odds of hypothyroidism within 4 of 7 American Kennel Club breed groups [35]. Similarly, the current study found that 5 of 7 UK Kennel Club breed groups had increased odds of hypothyroidism. Previously postulated theories to explain these associations included an explanation of increased likelihood for owners of purebred dogs to spend money on veterinary work-ups [34] and an increased likelihood for purebred dogs to have conditions detected during pre-breeding health screenings [35]. However, the present study identified that the purebred predisposition existed independently of insurance and neutering status, and therefore alternative genetic explanations may exist. It is possible that lower risk of hypothyroidism in crossbred dogs reflects some health-enhancing effects of hybrid vigour, whereby, although crossbred dogs can carry recessive alleles associated with disease, purebreds are more likely to be homozygous and therefore clinically affected due to more limited genetic diversity within their breed [36, 37]. Further studies are warranted to explore these possible effects.

Nine breeds in the current study showed significantly reduced odds (i.e., were protected) of having diagnosed hypothyroidism compared to crossbreds. Previously, only German shepherd dogs and crossbred dogs have been identified at reduced risk for hypothyroidism [8]. The current study identified reduced risk for the UK Kennel Club Toy and Terrier breed groups, although an earlier US study did not identify protections among The American Kennel Club Terrier and Toy breed groups [35]. The aetiology of these apparent protections from hypothyroidism in many small and toy breeds is unknown but could offer useful future avenues for research. Protected breeds may provide excellent control animals for genetic studies into hypothyroidism. Interestingly, Cavapoo or cavachon dogs did not show any cases of hypothyroidism in the current study, despite there being over 3,500 of each of these designer breeds in our cohort. Exploration of the apparent protective effect of creating “designer breeds” on reducing hypothyroidism and other disorders may allow breeding towards dogs with fewer predisposed conditions and an overall benefit on health and quality of life.

Dogs with a mean adult bodyweight at or above the breed/sex mean had 2.06 times the odds of diagnosed hypothyroidism compared to those with bodyweight below the breed mean in the current study. Although bodyweight relative to the breed/sex does not differentiate obese from larger framed lean dogs within their breed, dogs that weigh above the mean for their breed/sex are more likely to be overweight/obese [38]. Therefore, the current results support previous evidence that obesity or weight gain is present in 41–44% of dogs with hypothyroidism [4, 6] due to the metabolic effects of a lack of thyroid hormones. However, the temporal sequence of increased bodyweight and hypothyroidism diagnosis cannot be inferred from the current study; it is possible that larger and/or obese dogs have an increased risk for developing hypothyroidism rather than the reverse direction of causality [10]. In conclusion, with the nature of the clinical data used in the current study, it is not possible to fully determine cause and effect between increased bodyweight and hypothyroidism. In consequence, further studies are required to investigate the timeline for weight changes and hypothyroidism in dogs in more detail.

Neutered dogs of both sexes showed increased odds of diagnosed hypothyroidism in the current study, with the odds ratio being slightly higher for neutered females (OR 1.46) compared to neutered males (OR 1.31). However, the 95% confidence intervals overlapped between the entire animals of both sexes and between the neutered animals of both sexes, suggesting that sex per se is not associated with predisposition to hypothyroidism in dogs. Previous studies of hypothyroid dogs have reported conflicting information on sex and neuter status. One UK study did not identify an association of either variable with hypothyroidism [4] whilst a larger study of 3,206 dogs from US referral hospitals reported neutered females, but not neutered males, with increased risk compared to entire females [8]. A study of 66 hypothyroid dogs at a US University hospital reported higher relative risk for hypothyroidism in both neutered male and female dogs (OR 1.81 neutered males, OR 1.5 neutered females) [6]. It is possible that removal of sex steroids may impact the immune function and increase the risk of developing hypothyroidism. However, it is important to note that causality cannot be safely inferred from these studies because the timing of neutering was not considered. A large study of over 90,000 dogs at a US university teaching hospital reported that neutering at least 150 days prior to diagnosis was associated with hypothyroidism and other immune-mediated diseases [39], although the retrospective design of that study also prevented demonstration of causality. Cohort study designs that follow dogs over time are needed to more thoroughly explore the impact of neutering on the development of hypothyroidism [40].

Insured dogs had 2.27 times the odds of having diagnosed hypothyroidism compared to uninsured dogs. This supports previous assertions that veterinary care may be less accessed by owners of non-insured compared to insured animals and that non-insured animals may consequently have fewer medical procedures including diagnostics performed [41]. This finding highlights a potential canine welfare issue from constrained veterinary care funding for dogs without pet insurance. Previous studies based on primary care clinical records have similarly identified that insurance cover is associated with significantly increased odds of diagnosis for disorders in dogs (corneal ulceration OR 1.6 [42], chronic kidney disease OR 2.55 [43], patellar luxation OR 1.9 [44]) and cats (lymphoma OR 3.7 [45], hyperthyroidism OR 1.78 [46], diabetes mellitus OR 2.0 [47]. The current findings highlight potential welfare issues whereby there may be substantial numbers of dogs living with undiagnosed hypothyroidism in the wider population, and especially among the uninsured subset of dogs, and that clinicians should be aware of this phenomenon when evaluating patients in their clinics.

This study had some limitations related to the secondary use of veterinary clinical records for research that have been previously reported [48, 49]. These limitations include issues relating to missing data, poor standardization of diagnosis terms, variation in the rigor and quality of clinical notetaking, and variations in the levels of clinical care of animals achieved across owners and practices. In addition to these, previous epidemiological studies in dogs have been criticized for a lack of stringent criteria being applied to the case definition for diagnosis of hypothyroidism [2, 50]. No attempt was made in the current study to apply retrospective diagnostic criteria to confirm or exclude cases diagnosed as hypothyroid by the attending veterinary surgeon because we aimed to report on primary care veterinary caseloads as they are recognised and diagnosed rather than to cleanse these caseloads retrospectively based on external superimposed diagnostic criteria. It is also recognised that the attending veterinary teams would have access to, and considered, many additional diagnostic aids such as their personal history with the animal and the owner, the full presenting phenotype and their personal clinical experience that would be challenging to assess via the clinical records alone but that are recognised as being critical components of the evidence-based triad for diagnosis [33, 51]. Although this may have allowed inclusion of some misclassified cases, this big data study of a primary-care caseload aimed to reflect diagnostic protocols used in primary-care practice and to provide results with generalisability to the wider dog population. The current study reports the apparent prevalence of hypothyroidism in dogs (i.e. the proportion of dogs under veterinary care that are formally diagnosed) but this may be substantially different to the true prevalence value (i.e. the proportion of dogs with hypothyroidism if there were complete and accurate discernment across all dogs) [10, 43]. Application of pre-agreed diagnostic criteria (e.g. laboratory test cut-off values) may have assisted to retrospectively standardise the clinical decision-making across the large numbers of clinicians and practices in the study in an effort to reduce measurement bias in the current study [10]. The current study also included all cases (pre-existing and incident) within the risk factor analysis. Consequently, these results should be interpreted as showing the odds for being a hypothyroid case rather than the odds for becoming a hypothyroid case. In an ideal world, the current study would have explored associations between hypothyroidism and body condition score but unfortunately body condition score is not recorded consistently across primary care clinical records; for this reason, the current study explored associations with mean adult bodyweight instead.

## Conclusion

This study examined a large sample of dogs with diagnosed hypothyroidism under primary veterinary care in the UK. The primary-care prevalence of diagnosed hypothyroidism was similar to other common canine endocrinopathies including diabetes mellitus and Cushing’s syndrome. Strong predispositions and protections for diagnosed hypothyroidism were identified in several breeds. Several other risk factors were also identified that can help veterinary surgeons during clinical work-up of suspected cases. Identification of novel evidence for protected breeds provides useful information for research into genetic mechanisms.

## Data Availability

The datasets generated during and/or analysed during the current study will be made available at the RVC Research Online repository.

## Abbreviations

CI: Confidence interval
IQR: Interquartile range
OR: Odds ratio
